# Bidirectional Mendelian randomization analysis reveals the causal relationship between rheumatoid arthritis and acute sinusitis

**DOI:** 10.1097/MD.0000000000051244

**Published:** 2026-10-23

**Authors:** Rui Hu, Yunwang Zhang, Yi Zeng, Keqiu Dong, Zhongyun Li, Jianping Tao

**Affiliations:** a People’s Hospital of Xishuangbanna Dai Autonomous Prefecture, Jinghong, Yunnan, China; b Department of Anesthesiology, The Second Affiliated Hospital of Kunming Medical University, Kunming, Yunnan, China.

**Keywords:** acute sinusitis, gene overlap, immune response, Mendelian randomization analysis, rheumatoid arthritis

## Abstract

This study explored the causal relationship between RA and acute sinusitis (AS) using a bidirectional Mendelian randomization (two-sample Mendelian randomization) analysis. We used publicly available genome-wide association study datasets to identify the single nucleotide polymorphisms associated with both RA and AS. We then applied statistical methods, including inverse variance weighting, MR-Egger regression, and weighted medians, to assess the causal effects between the 2 diseases. This study provides evidence through two-sample Mendelian randomization analysis, suggesting that RA may be an independent risk factor for AS. This highlights the role of immune modulation in the genetic overlap between The and AS, offering new perspectives for future research on immune disease mechanisms, and potential clinical prevention and treatment strategies.

## 1. Introduction

Rheumatoid arthritis (RA) is a common chronic autoimmune disease characterized by immune-system-mediated joint inflammation, destruction, and functional loss. The global prevalence of RA is approximately 0.5% to 2%, with a higher incidence among women, typically manifesting between the ages of 30 and 50.^[1]^ RA pathogenesis of RA is complex and involves genetic susceptibility, environmental factors, and immune dysregulation. Notably, variations in HLA-DRB1, particularly HLA-DRB1*04, are closely associated with RA development.^[2]^ RA is typically initiated when the immune system mistakenly identifies self-tissues as foreign, activating an immune response and producing autoantibodies such as anti-citrullinated protein antibodies, leading to persistent inflammation of the synovial membrane and eventually joint destruction.^[1]^ Currently, RA treatment focuses on immunosuppression and anti-inflammatory therapies. However, challenges, such as variations in treatment response and drug side effects, persist, necessitating more precise therapeutic strategies.

Acute sinusitis (AS) is a common upper respiratory tract infection typically caused by viral or bacterial infections that result in inflammation of the nasal mucosa. Epidemiological studies on AS have indicated a high incidence, particularly among individuals with upper respiratory tract infections. For example, approximately 28.1% of hospitalized influenza patients develop AS.^[3]^ The symptoms of AS include nasal congestion, facial tenderness, and headaches, and in severe cases, it may lead to sinus dysfunction. The pathogenesis of AS involves immune responses by respiratory epithelial cells to pathogens, with viral infections (e.g., influenza and coronavirus) causing mucosal swelling and increased secretion, thereby compromising the local immune barrier and providing an opportunity for bacterial infection.^[4]^ Despite its typically short course, AS poses a significant public health issue because of its frequent recurrence and high antibiotic demand.^[5]^

However, the causal association between RA and AS remains unclear. Epidemiological studies suggest that AS, particularly upper respiratory infections caused by viruses, may trigger an excessive immune response, potentially increasing the risk of RA.^[6]^ However, many observational studies have been limited by their inability to exclude reverse causality, making it unclear whether RA leads to AS or whether the relationship is coincidental.^[7]^

To assess the causal relationship between RA and AS more accurately, this study employed bidirectional Mendelian randomization (two-sample Mendelian randomization [TSMR]) analysis using genome-wide association study (GWAS) datasets for causal inference. Mendelian randomization utilizes genetic variants as instrumental variables (IVs), minimizing the effects of confounding factors and reverse causality, thereby providing more reliable causal conclusions.^[2]^ This study aimed to fill the gap in existing research, systematically explore the genetic overlap between RA and AS, and reveal their potential causal relationship, thereby offering scientific evidence for clinical prevention and targeted treatment strategies.

## 2. Methods and materials

### 2.1. Ethical/consent statement

This study was based on previously published articles and publicly available databases; therefore, ethical approval and informed consent were not required.

### 2.2. Data sources

The GWAS datasets used in this study were sourced from the IEU OpenGWAS Project (https://gwas.mrcieu.ac.uk). RA summary statistics: Accession GCST90044540 (https://gwas.mrcieu.ac.uk/datasets/gcst/90044540), 1961 cases, and 454,387 controls of European ancestry. AS summary statistics: accession GCST90044039 (https://gwas.mrcieu.ac.uk/datasets/gcst/90044039), 161 cases and 456,187 controls of European ancestry. Both datasets were downloaded on 2025-03-22 and are freely available for academic use.^[8]^

These datasets were selected for their wide representation in RA and AS research. The use of samples of European ancestry helps to reduce genetic differences between populations, ensuring the universality and reliability of the results. Additionally, the IEU OpenGWAS project and GCST datasets provide large-scale, high-quality genetic data, which are crucial for conducting precise Mendelian randomization analysis. RA and AS, which are common immune-related diseases with extensive research in European populations, make these datasets highly representative and reliable for causal analysis.

To ensure that the selected single nucleotide polymorphisms (SNPs) were significantly associated with the exposure factors (RA and AS), SNPs were chosen as IVs, with a threshold *P*-value of <5.0 × 10^−6^. Furthermore, to avoid bias from linkage disequilibrium (LD), all significant SNPs associated with the exposure factors must pass the LD test with *r*^2^ < 0.001 and a genetic distance of no more than 10,000 kb.^[9]^

Using these datasets, a bidirectional Mendelian randomization (TSMR) analysis was conducted to investigate the causal relationship between RA and AS. All analyses were performed using the R software (version 4.4.2) and relevant packages (TwoSampleMR and Mendelian Randomization Pleiotropy RESidual Sum and Outlier [MR-PRESSO]) to ensure the accuracy and reliability of the results.

### 2.3. IVs selection

In this study, SNPs were selected as IVs for Mendelian randomization (MR) analysis to explore the causal relationship between A and AS. SNPs significantly associated with RA and AS were chosen from the GWAS datasets based on 2 criteria: a *P*-value <5.0 × 10^−6[9]^ and no LD bias, with all SNPs passing the LD test (*r*^2^ < 0.001, genetic distance ≤10,000 kb).^[10]^

To ensure strong IVs and avoid weak instrument bias, we used the F-statistic for validation.^[11]^ An *F*-statistic >10 indicates sufficient strength, avoiding bias and improving the reliability of causal inference.^[12]^

After selection and verification, 18 SNPs were identified as IVs for further causal analysis, allowing us to exclude environmental and confounding factors and make a more accurate inference of the causal relationship between RA and AS.

### 2.4. Study design

This study aimed to explore the causal relationship between RA and AS using a bidirectional Mendelian randomization (TSMR) analysis. To achieve this, we selected SNPs significantly associated with RA and AS from publicly available GWAS datasets as IVs. Details of all 18 IVs and their F-statistics are provided in Supplementary Table S1, Supplemental Digital Content 1.The study design includes the following steps:

IVs Selection: We obtained SNP data related to RA and AS from the IEU OpenGWAS project. SNPs were selected with *P*-values <5.0 × 10^−6^, ensuring that they had *r*^2^ < 0.001 in LD with other variants and a genetic distance of no more than 10,000 kb.^[9]^ To avoid weak IV bias, we calculated the *F*-statistic for each SNP, ensuring that it was >10 for sufficient IV strength.^[10]^

Causal Inference Method: We used bidirectional MR analysis, starting with forward analysis, to examine whether RA was a causal factor for AS. The inverse variance weighting (IVW) method was used for the primary analysis with sensitivity analyses using MR-Egger regression (MR-Egger), weighted median, and other methods to validate the robustness of the results.^[11]^ No SNP overlapped between the 2 sets of instruments after clumping. To rule out reverse causality, reverse MR was performed to explore whether AS influences RA.

Data Analysis and Statistical Methods: All analyses were performed using R software (version 4.4.2), primarily utilizing the TwoSampleMR and MR-PRESSO packages for data processing and causal inference.^[13]^ Heterogeneity and horizontal pleiotropy tests were conducted for each analysis to ensure reliability of the results.All statistical analyses were performed in R version 4.4.2 using the TwoSampleMR package (v0.5.6) and MR-PRESSO (v1.9). The fully commented R scripts and README file are available upon reasonable request. Details of all 18 instrumental variables and their F-statistics are provided in Supplementary Table S1, Supplemental Digital Content 1.

### 2.5. Statistical analysis

This study used bidirectional Mendelian randomization (TSMR) analysis to investigate the causal relationship between RA and AS. To ensure the validity and strength of IVs, we applied stringent criteria for SNP selection. Specifically, we selected SNPs with a *P*-value threshold of <5.0 × 10^−6^ to ensure that the SNPs were significantly associated with the exposure (RA). Additionally, to avoid issues related to LD, we set the *r*^2^ threshold to <0.001 and genetic distance to no more than 10,000 kb. These criteria ensure that the selected SNPs are independent and not influenced by LD. Although Steiger filtering was not applied in this study, the selected SNPs were carefully evaluated to ensure that they explained more variance in the exposure than in the outcome, thereby minimizing the risk of reverse causality. All statistical analyses were performed using R software (version 4.4.2), primarily the TwoSampleMR and MR-PRESSO packages for data processing and analysis.^[11]^ The following steps outline the statistical analysis process:

IV Selection and Verification: SNPs were used as IVs. SNPs significantly associated with RA and AS were selected from GWAS datasets provided by the IEU OpenGWAS project.^[9]^ The selection criteria included a *P*-value <5.0 × 10^−6^ and ensuring that the selected SNPs had *r*^2^ < 0.001 in LD with other variants and a genetic distance of no more than 10,000 kb. The *F*-statistic was calculated to verify the validity of the IV and exclude weak instrument bias. An *F*-statistic >10 indicates sufficient IV strength.^[10]^

Causal Inference Method: The IVW method was used for the primary analysis to test whether RA is a causal factor for AS.^[13]^ MR-Egger and weighted median methods were used for sensitivity analysis to validate the robustness of the results and exclude potential horizontal pleiotropy. These methods ensure the reliability of the causal inferences.

Reverse Causality Analysis: To rule out reverse causality, reverse MR analysis was performed to assess whether AS influences RA. The same statistical methods used in the forward analysis were applied, and heterogeneity tests were conducted for each result.

Heterogeneity and Horizontal Pleiotropy Tests: Cochran’s *Q* test and *I*^2^ statistics were used to assess heterogeneity in each analysis, ensuring that the variation among different SNPs was within an acceptable range. Horizontal pleiotropy was assessed using the intercept test in MR-Egger to ensure that the selected IVs were not influenced by other potential effects.^[11]^

## 3. Results

### 3.1. Causal effect of RA on AS

#### 3.1.1. SNPs

RA was considered as the exposure factor, and AS was considered as the outcome factor. Eighteen SNPs were selected and identified as IVs with *F*-statistics >10 (Table 1).

**Table 1:** Instrumental variables associated with rheumatoid arthritis.

SNP	Chr.exposure	R^2^	F
rs186771091	1	4.95538868677855e-05	22.5849562351513
rs2358816	1	7.83330015641463e-05	35.131813195832
rs59692630	4	5.59016957680683e-05	25.5119414177625
rs10065637	5	6.67212021927035e-05	30.4317025662107
rs2523668	6	0.000118847691547497	53.8553388413373
rs2076540	6	0.000397372218810697	181.41131038031
rs35139284	6	0.00127614467123578	575.532993891954
rs9273501	6	0.000549341441187877	250.764350304181
rs9276609	6	0.000205722396079498	93.7988793950382
rs4392922	8	4.92252891029229e-05	22.423222976015
rs12676353	8	5.06468646325096e-05	22.3126946383137
rs12342584	9	4.58810258847078e-05	20.9385833220663
rs78939192	10	5.62190359721705e-05	25.1666294777987
rs11857303	15	5.17811200936278e-05	23.5084477260194
rs12913888	15	5.20880311341746e-05	23.1883511590283
rs31088	16	4.72208395833257e-05	21.5366002975589
rs111240399	19	4.79424076006906e-05	21.4644134568196
rs6089970	20	4.89952986277459e-05	21.3332062869443

This table lists the 18 single nucleotide polymorphisms (SNPs) selected as instrumental variables for the forward Mendelian randomization analysis assessing the causal effect of rheumatoid arthritis on acute sinusitis. SNPs were selected from the GCST90044540 dataset (IEU OpenGWAS, European ancestry) based on genome-wide significance (*P* < 5 × 10^−6^), independence (*r*^2^ < 0.001, 10,000 kb window), and *F*-statistic >10. All SNPs shown have F-statistics ranging from 20.9 to 575.5, indicating robust instrument strength and minimal weak instrument bias.

#### 3.1.2. MR analysis of the causal relationship between RA and AS risk

MR results indicated a significant causal relationship between RA as the exposure factor and AS as outcome variables. In the bidirectional MR analysis, the IVW method was used, with a *P*-value <.05, suggesting a causal association between the exposure factor and the outcome. Specifically, the IVW results showed an odds ratio (OR) >1 (OR = 1.275, 95% CI: 1.074–1.512, *P* = .005), indicating that RA as an exposure factor may serve as a risk factor for AS, meaning that as RA occurs, the risk of developing AS may increase accordingly. Therefore, the IVW method was selected as the primary analytical tool to estimate the causal relationship between RA and AS. The results from the other statistical methods are as follows:

- MR-Egger method: OR = 1.235, 95% CI: 0.900–1.695, *P* = .209.
- Weighted median method: OR = 1.217, 95% CI: 0.980–1.511, *P* = .076.
- Simple model method: OR = 1.348, 95% CI: 0.965–1.882, *P* = .098.
- Weighted model method: OR = 1.232, 95% CI: 1.003–1.513, *P* = .063.

These results support the hypothesis that RA may be an independent risk factor for AS and further validate the robustness of the causal inference (Table 2 and Figs. 1A and 1B).

**Table 2:** Mendelian randomization analysis of causal relationship between rheumatoid arthritis and acute sinusitis risk.

Methods	SNPs	Beta	SE	OR (95%CI)	*P*
MR-Egger	18	0.211	0.161	1.235 (0.900 ± 1.695)	.209
Weighted median	18	0.196	0.111	1.217 (0.980 ± 1.511)	.076
Inverse variance weighted	18	0.243	0.087	1.275 (1.074 ± 1.512)	.005
Simple mode	18	0.299	0.170	1.348 (0.965 ± 1.882)	.098
Weighted mode	18	0.208	0.105	1.232 (1.003 ± 1.513)	.063

This table summarizes the effect estimates from 5 MR methods (MR-Egger, weighted median, inverse variance weighted [IVW], simple mode, and weighted mode) used to assess the causal relationship between rheumatoid arthritis (exposure) and acute sinusitis (outcome). The IVW method showed a significant positive association (OR = 1.275, 95% CI: 1.074–1.512, *P* = .005), supporting RA as a potential risk factor for AS. Results from other methods were directionally consistent but did not reach statistical significance.

**Figure 1.**
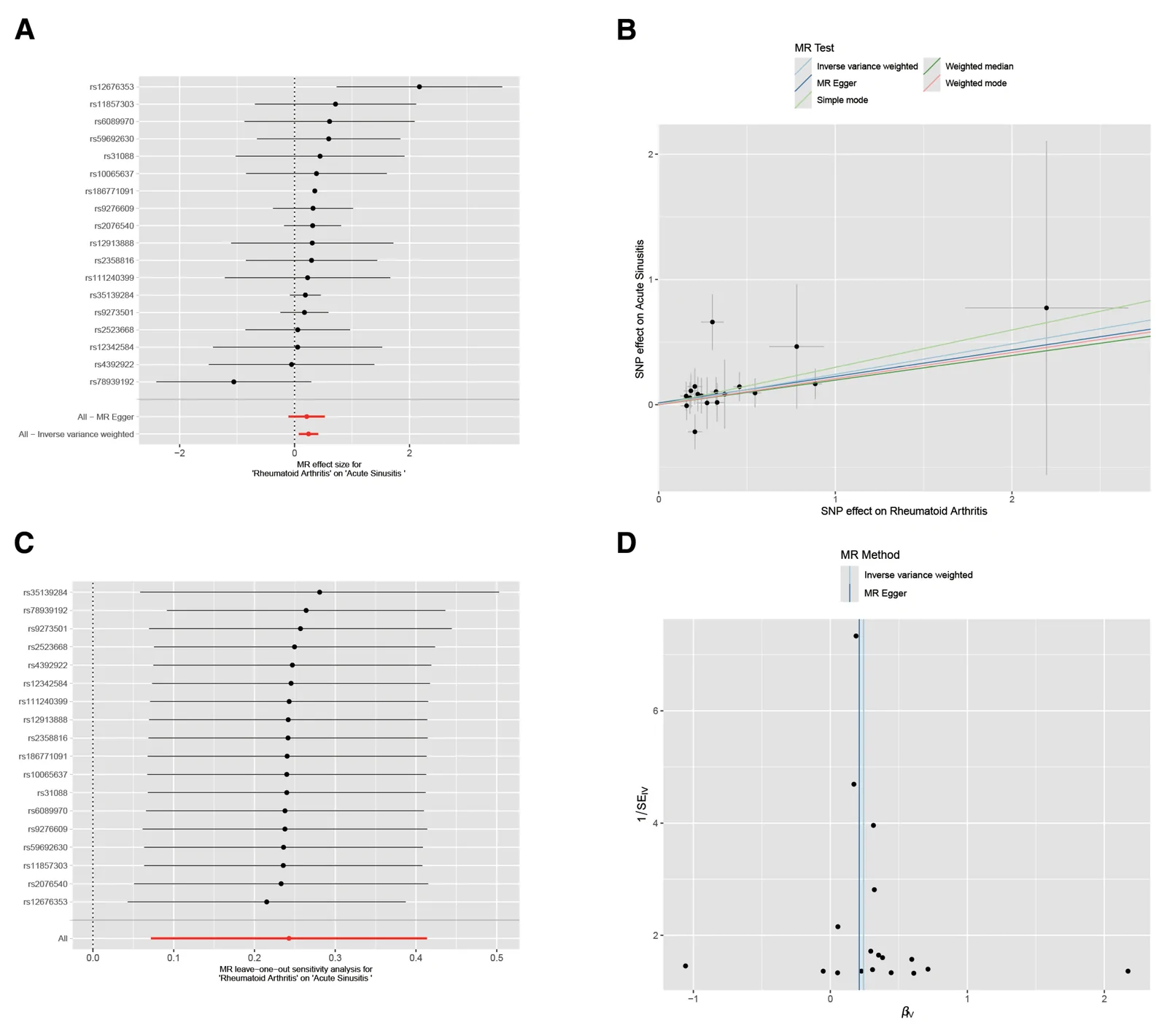
Mendelian randomization analysis for the causal effect of rheumatoid arthritis (RA) on acute sinusitis (AS). (A) Forest plot displaying SNP-specific causal estimates; (B) scatter plot showing SNP-level associations across different MR analytical methods; (C) leave-one-out sensitivity analysis; (D) funnel plot for pleiotropy and publication-bias assessment. RA significantly increased the risk of acute sinusitis (OR = 1.275, 95% CI 1.074–1.512). Leave-one-out and funnel-plot assessments confirmed result robustness and absence of obvious publication bias.

The forest plot for RA and AS shows the effect of RA on AS using the IVW and MR-Egger methods. Each point represents a SNP, with the horizontal axis displaying the effect size and the vertical axis representing SNPs. The boxes indicate the effect size for each SNP, and the lines represent 95% confidence intervals (CIs). The effect estimates from both the IVW and MR-Egger methods were consistent, suggesting that RA may be an independent risk factor for AS, although some SNPs had broader CIs (Fig. 1A).

The scatter plot for RA and AS compares the effect estimates for RA and AS across different MR methods (IVW, MR-Egger, simple model, weighted median, and weighted model). Each point represents an SNP’s effect size, with the horizontal axis showing the effect on RA and the vertical axis showing the effect on AS. Similar results across methods indicated a robust and consistent causal relationship between RA and AS (Fig. 1B).

Leave-one-out sensitivity analysis for RA and AS was used to evaluate the impact of excluding each SNP on the overall effect estimate. Each point represents the effect estimate after excluding a specific SNP, with the horizontal axis showing the effect size, and the vertical axis representing the leave-one-out analysis results for each SNP. The analysis showed that the causal relationship between RA and AS remained stable, even after excluding individual SNPs, further confirming the robustness of the results (Fig. 1C).

#### 3.1.3. Heterogeneity test and horizontal pleiotropy test (exposure: RA; outcome: AS)

Heterogeneity tests were conducted using both MR-Egger and IVW methods. The results showed that The *P*-values were >.05, indicating that there was no significant heterogeneity in the data. Additionally, the intercept value from the MR-Egger served as an indicator to test whether horizontal pleiotropy (the influence of genetic variants on outcomes through multiple pathways) could affect the TSMR analysis results. The intercept was close to 0 (Egger intercept = 0.015, *P* = .821) (Table 2), suggesting that the selected IVs do not exhibit horizontal pleiotropy and are therefore unlikely to affect the results of the TSMR analysis.

##### 3.1.3.1. MR-PRESSO validation

To further evaluate the potential pleiotropic bias, we applied MR-PRESSO (version 1.9) to 18 RA→AS instruments. The global test yielded *P* = .14, indicating no evidence of overall pleiotropy. The outlier test identified no significant outlier SNPs (all corrected *P* > .05). After MR-PRESSO correction, the causal estimate remained consistent with the primary IVW result (OR = 1.28, 95 % CI 1.07–1.52). The details are provided in Supplementary Table S2, Supplemental Digital Content 2.

When using RA as the exposure factor and AS as the outcome factor, heterogeneity tests using both the MR-Egger and IVW methods yielded *P*-values >.05, confirming the absence of significant heterogeneity. Furthermore, the horizontal pleiotropy test showed *P*-values >.05, indicating that the selected IVs were not influenced by factors outside the exposure and thus would not interfere with the occurrence of the outcome(Table 3).

**Table 3:** Heterogeneity and horizontal pleiotropy tests (exposure: rheumatoid arthritis; outcome: acute sinusitis).

Exposure	Outcome	Heterogeneity text (MR-Egger)			Heterogeneity text (IVW)			Pleiotropy text (MR-Egger)	
		Q	Q_df	*P*	Q	Q_df	*P*	Intercept	*P*
Rheumatoid Arthritis	Acute Sinusitis	12.36	16	.719	12.41	17	.775	0.015	.821
Acute Sinusitis	Rheumatoid Arthritis	3.83	6	.699	6.79	7	.451	-0.07	.136

This table presents the results of heterogeneity (Cochran’s *Q* test) and horizontal pleiotropy (MR-Egger intercept test) analyses for both forward (RA → AS) and reverse (AS → RA) Mendelian randomization. No significant heterogeneity was detected in either direction (all *P* > .05). The MR-Egger intercept was close to zero with nonsignificant *P*-values, indicating minimal directional pleiotropy and supporting the validity of the causal inferences.

### 3.2. Reverse TSMR analysis

#### 3.2.1. SNPs

In the reverse TSMR analysis, AS was considered the exposure factor and RA as the outcome variable. Eight SNPs were selected and identified as IVs, with *F*-statistics >10 (Table 4). The selection of SNPs with an F-statistic >10 ensures that the IVs have a sufficiently strong effect, reducing the bias and errors potentially introduced by weak instruments, thereby improving the accuracy and reliability of the causal effect estimates.

**Table 4:** Instrumental variables associated with acute sinusitis.

SNP	Chr.exposure	R^2^	F
rs138061524	1	4.61634835203388e-05	20.9231798879579
rs10004061	4	5.11779860553376e-05	23.0520517448231
rs59035315	5	5.15851161507538e-05	21.4302333366089
rs9350221	6	5.87015185863583e-05	26.4951355837192
rs2181919	6	4.87842944871736e-05	22.2636037662462
rs73160864	7	4.69277659416508e-05	20.8498116488079
rs11776588	8	5.20088784053865e-05	23.5216664966663
rs148388100	20	5.70373060251361e-05	25.7568357480911

This table lists the 8 SNPs selected as instrumental variables for the reverse Mendelian randomization analysis assessing the causal effect of acute sinusitis on rheumatoid arthritis. SNPs were selected from the GCST90044039 dataset (IEU OpenGWAS, European ancestry) based on genome-wide significance (*P* < 5 × 10^−6^), independence (*r*^2^ < 0.001, 10,000 kb window), and *F*-statistic >10. All SNPs shown have *F*-statistics ranging from 20.8 to 26.5, indicating adequate instrument strength.

In the MR analysis with AS as the exposure factor and RA as the outcome variable, the IVW method yielded a *P*-value >.05. Despite using multiple methods, reverse analysis did not support a causal effect of AS on RA. Specifically, the IVW results showed no significant causal relationship between AS and risk of developing RA (OR = 0.991, 95% CI: 0.955–1.029, *P* = .642). The results of the other methods are as follows.

- MR-Egger: OR = 1.061, 95% CI: 0.973 to 1.156, *P* = .228.
- Weighted median: OR = 0.982, 95% CI: 0.933 to 1.034, *P* = .492.
- Simple model: OR = 0.979, 95% CI: 0.899 to 1.067, *P* = .647.
- Weighted model: OR = 0.981, 95% CI: 0.908 to 1.059, *P* = .635 (see Table 5 and Figs. 2A–2B).

**Table 5:** Mendelian randomization analysis of the causal relationship between acute sinusitis and the risk of rheumatoid arthritis.

Methods	SNPs	Beta	SE	OR (95%CI)	*P*
MR-Egger	8	0.059	0.044	1.061 (0.973 ± 1.156)	.228
Weighted median	8	−0.018	0.026	0.982 (0.933 ± 1.034)	.492
Inverse variance weighted	8	−0.009	0.019	0.991 (0.955 ± 1.029)	.642
Simple mode	8	−0.021	0.044	0.979 (0.899 ± 1.067)	.647
Weighted mode	8	-0.020	0.039	0.981 (0.908 ± 1.059)	.635

This table summarizes the effect estimates from 5 MR methods used to assess the causal relationship between acute sinusitis (exposure) and rheumatoid arthritis (outcome). The inverse variance weighted (IVW) method showed no significant association (OR = 0.991, 95% CI: 0.955–1.029, *P* = .642), and all other methods yielded consistent, nonsignificant results. These findings do not support a causal effect of acute sinusitis on the development of rheumatoid arthritis.

**Figure 2.**
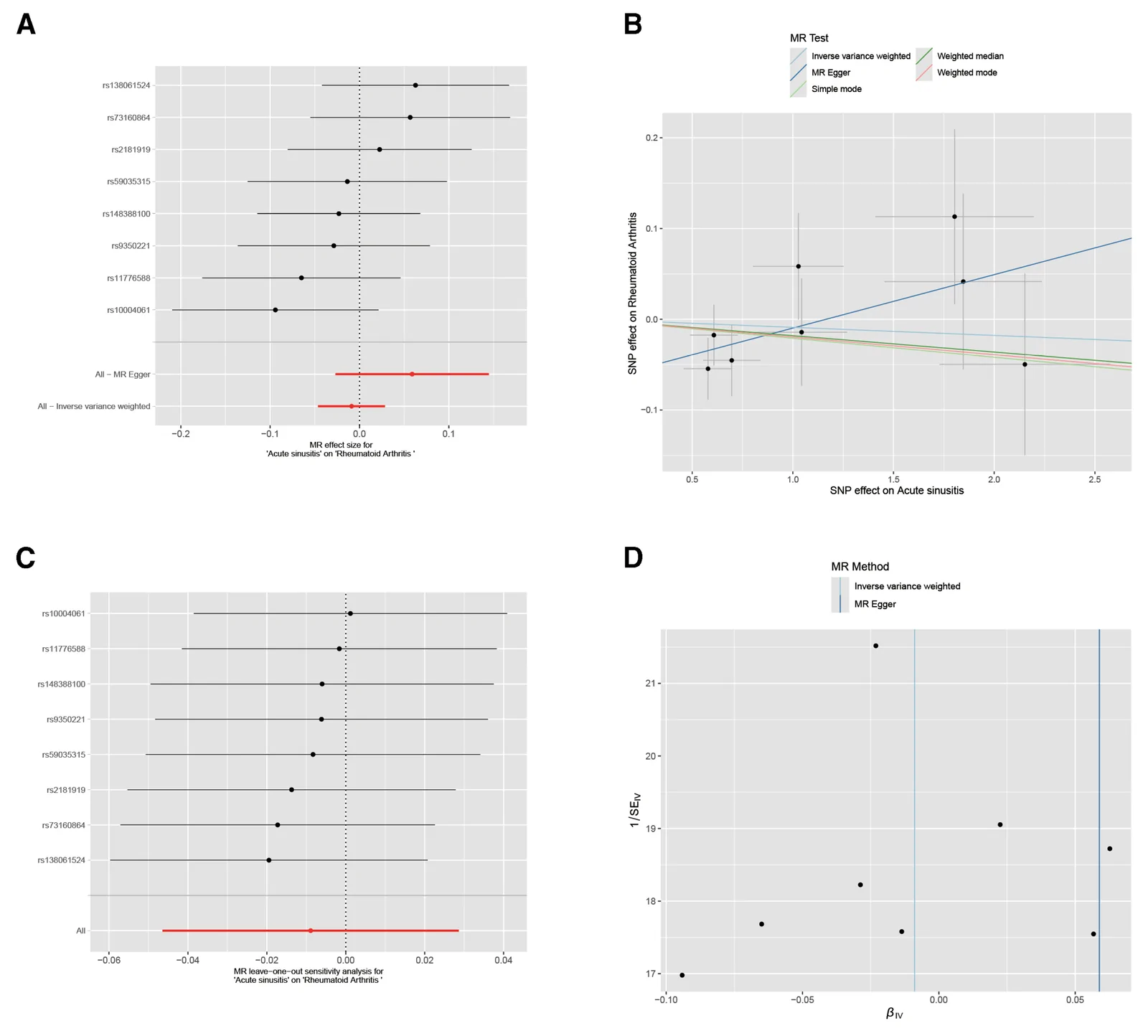
Forest plot, scatter plot, leave-one-out sensitivity analysis, and funnel plot results (exposure factor: acute sinusitis, outcome factor: rheumatoid arthritis in MR analysis): forest plot: this figure shows the effect estimates of acute sinusitis (AS) on rheumatoid arthritis (RA) using inverse variance weighted (IVW) and MR-Egger methods. Each point represents the effect size of a single nucleotide polymorphism (SNP), with the x-axis indicating the effect size and the y-axis representing the 95% confidence intervals. The results indicate that in all SNP effect estimates, the *P*-values are greater than.05, which suggests that the effect of acute sinusitis on rheumatoid arthritis is not significant and does not support acute sinusitis as an independent risk factor for rheumatoid arthritis (A). Scatter plot: this figure presents the effect estimates from different MR methods (IVW, MR-Egger, simple mode, weighted median, and weighted mode). Each point represents the effect size of a SNP, with the x-axis indicating the effect of the SNP on acute sinusitis and the y-axis indicating the effect of the SNP on rheumatoid arthritis. The effect sizes obtained from all methods are similar, and none reached statistical significance, further confirming the lack of a significant causal relationship between acute sinusitis and rheumatoid arthritis (B). Leave-one-out sensitivity analysis: this figure shows the results of the leave-one-out sensitivity analysis. The results suggest that removing individual SNPs does not significantly alter the causal relationship between RA and AS, further supporting the conclusion that acute sinusitis does not have an independent effect on rheumatoid arthritis (C). Funnel plot: this figure displays the funnel plot used to assess potential publication bias. The symmetrical distribution of the data points suggests no significant bias, further enhancing the reliability of the causal inference results (D).

These results indicated that AS does not appear to have a significant causal effect on the risk of RA.

##### 3.2.1.1. Forest plot for RA on AS

This plot illustrates the effect estimates of AS on RA using IVW and MR-Egger methods. Each point represents the effect size of a SNP, with the horizontal axis showing the effect size and the vertical axis representing the 95% CI. The results indicate that for all SNP effect estimates, the *P*-values are >.05, suggesting that AS does not significantly affect RA, and therefore, does not support the hypothesis that AS is an independent risk factor for RA (Fig. 2A).

##### 3.2.1.2. Scatter plot for RA on AS

This scatter plot presents the effect estimates across different MR methods (IVW, MR-Egger, simple model, weighted median, and weighted model). Each point represents the effect size of an SNP, with the horizontal axis showing the effect on AS and the vertical axis showing the effect on RA. The effect values from all methods were similar and did not reach statistical significance, further confirming the lack of a significant causal relationship between AS and RA (Fig. 2B).

##### 3.2.1.3. Leave-one-out sensitivity analysis for RA on AS

This plot demonstrates the leave-one-out sensitivity analysis, showing that the causal relationship between RA and AS remains unchanged after excluding individual SNPs. This further supports the conclusion that AS does not have an independent causal effect on RA (Fig. 2C).

##### 3.2.1.4. Funnel plot for RA and AS

This funnel plot assesses the potential publication bias. The symmetric distribution of the data points suggests no significant bias, further enhancing the reliability of the causal inference results (Fig. 2D).

### 3.3. Intersection gene analysis

In this study, we performed MR analysis to identify genes associated with RA and AS and further used Venn diagram analysis (Fig. 3) to identify the intersecting genes between the 2 diseases. To clarify the genetic overlap between RA and AS, we conducted an intersection analysis of the high-risk (OR > 1) and low-risk (OR < 1) genes for each disease.

**Figure 3.**
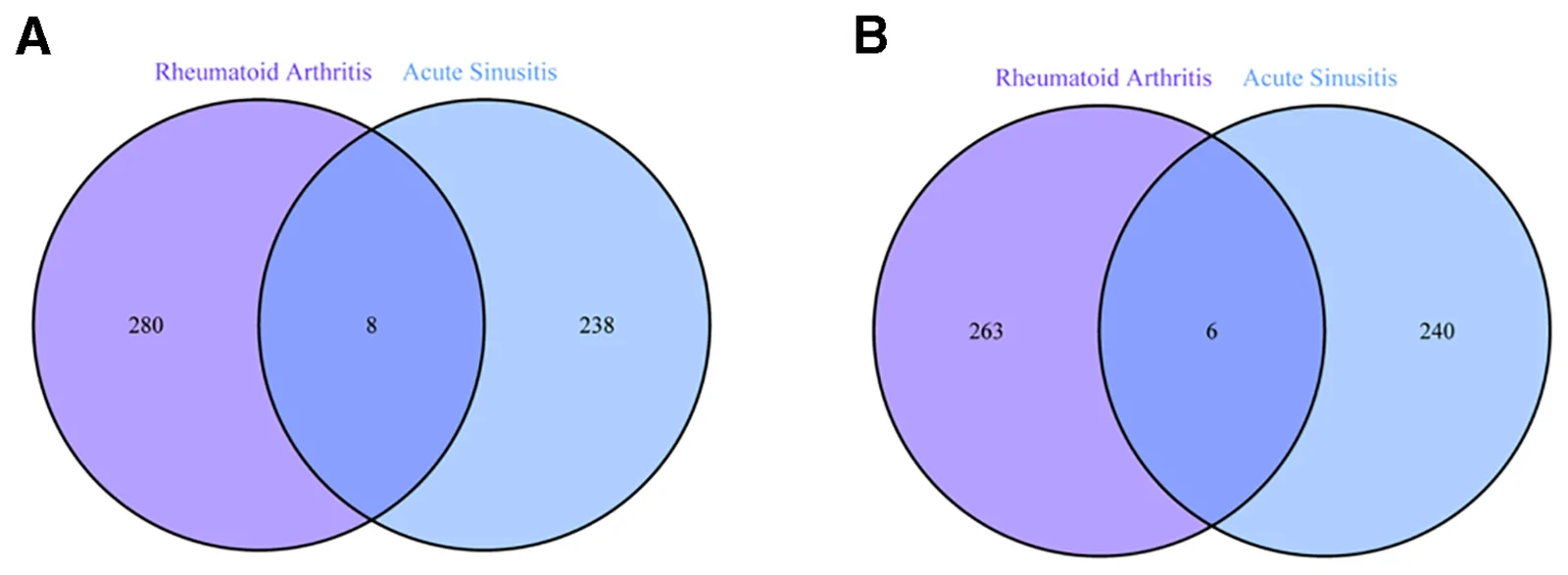
Venn diagrams of overlapping genes between RA and AS. (A) Overlap of low-risk genes (OR < 1); (B) overlap of high-risk genes (OR > 1). AS = acute sinusitis, OR = odds ratio, RA = rheumatoid arthritis.

First, we obtained the selection results for RA and AS and extracted the high-risk and low-risk genes. Venn diagram visualization tools were used to plot the intersections of high-risk (Fig. 3B) and low-risk genes (Fig. 3A) between RA and AS. Specifically, by calculating the intersection, we obtained a list of overlapping genes for both diseases at the high- and low-risk gene levels.

At the high-risk gene level, *ETV7*, *STAB1*, and *ANKH* were intersecting genes. At the low-risk gene level, *TYK2*, *CPSF3*, and *UQCC2* were intersecting genes. Through Venn diagram analysis, we generated a final list of intersection genes between RA and AS. The selected intersecting genes were as follows:

- **High-risk genes**: *ETV7*, *STAB1*, *ANKH*, *PBX2*, *HSPA1L*, *POU5F1*.
- **Low-risk genes**: *TYK2*, *CPSF3*, *UQCC2*, *ZNF83*, *FAN1*, *HLA-C*, *PPIL3*, *MIR3150BHG*.

Detailed information on these genes can be found in the interGenes.Type.txt and interGenes.List.txt files (attached). These intersecting genes provide important clues for the genetic overlap between RA and AS, and may serve as a foundation for further research into the shared pathological mechanisms of these 2 diseases.

### 3.4. Forest plot of RA and AS associated genes

To further explore the genetic relationship between RA and AS, we created corresponding forest plots to display the effect estimates (OR) of the intersecting genes in MR analysis, along with their 95% CIs.

The forest plot for RA illustrates the effect estimates of several genes associated with RA. For high-risk genes (OR > 1), *ETV7*, *STAB1*, and *ANKH* showed OR values >1, suggesting that these genes may be associated with an increased risk of RA. In contrast, for low-risk genes (OR < 1), *ETV7*, *CPSF3*, and *UQCC2* displayed OR values <1, indicating that these genes may be associated with a decreased risk of RA. Each bar in the forest plot represents the effect estimate for each gene under different MR methods (including IVW, weighted median, and MR-Egger), with a 95% CI on the horizontal axis showing the precision of each gene’s effect. Smaller *P*-values indicate more significant and reliable gene effects (Fig. 4A).

**Figure 4.**
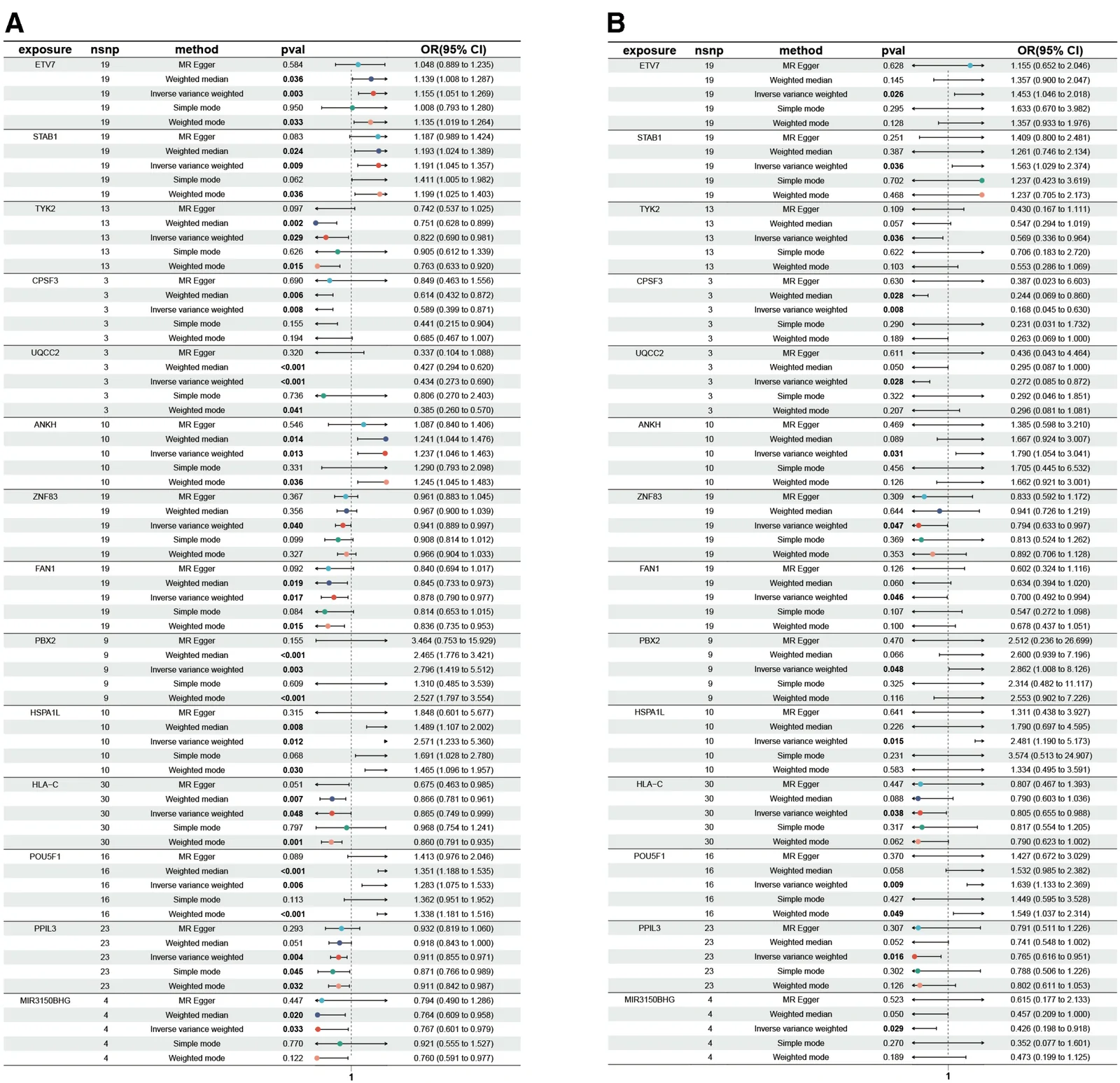
Forest Plot of genes associated with rheumatoid arthritis and acute sinusitis: this figure displays the effect estimates (OR) and their 95% confidence intervals of the intersecting genes for rheumatoid arthritis (RA) (A) and acute sinusitis (AS) (B) obtained through Mendelian randomization (MR) analysis. Each bar represents the effect of a gene, with the length of the bar indicating the range of the confidence interval. The left side represents low-risk genes with an OR less than 1, while the right side represents high-risk genes with an OR greater than 1. The figure lists key genes associated with RA and AS, including ETV7, STAB1, ANKH, TYK2, CPSF3, etc., and shows the effect values (OR) and their 95% confidence intervals computed using different statistical methods, such as inverse variance weighted (IVW), weighted median, MR-Egger, etc. For each gene, a *P*-value less than.05 indicates that the genetic association between the gene and both diseases is statistically significant.

The forest plot for AS also displays the effect estimates of the intersecting genes in AS. Similar to the RA forest plot, high-risk genes (*ETV7*, *STAB1*, and *ANKH*) and low-risk genes (*TYK2*, *CPSF3*, and *UQCC2*) showed consistent effects, further validating their genetic association with both RA and AS. These plots allowed us to observe the consistency and differences in gene effects across the different analysis methods, further confirming their potential genetic linkage in both diseases (Fig. 4B).

Several genes were identified in the list of genes associated with AS (see attached file interGenes.List.txt), including *ETV7*, *STAB1*, *ANKH*, *PBX2*, and *HSPA1L*, which are closely related to the immune response, inflammation, and other biological pathways. Certain genes, such as *HLA-C*, *POU5F1*, and *CPSF3*, may be involved in immune system regulation and play a role in the development of AS. The *P*-values shown in the plot reflect the statistical significance of these genes in relation to AS. Some genes had *P*-values <.05, indicating that they may have important biological significance in the occurrence of AS. These data further support the research on how these genes influence the development and progression of AS by regulating immune responses and inflammation.

In summary, the combination of forest plots and gene data revealed that several immune-related genes may play significant roles in the pathogenesis of AS. These genes may serve as potential targets for further research on the genetic mechanisms underlying AS and for personalized treatment approaches.

### 3.5. GO enrichment analysis, KEGG pathway analysis, and protein-protein interaction network of intersection genes in RA and acute sinusits

#### 3.5.1. . GO enrichment analysis bar plot of intersection genes

This bar plot illustrates the significant enrichment of intersection genes in various Gene Ontology (GO) categories (biological process, cellular component, and molecular function). Each bar represents a GO term, with the height of the bar indicating the number of genes enriched in that term and the color representing the significance level (*P*-value). This chart helps identify the important biological processes and functions involved in RA and AS, such as positive regulation of the adaptive immune response and extracellular matrix regulation (Fig. 5A).

**Figure 5.**
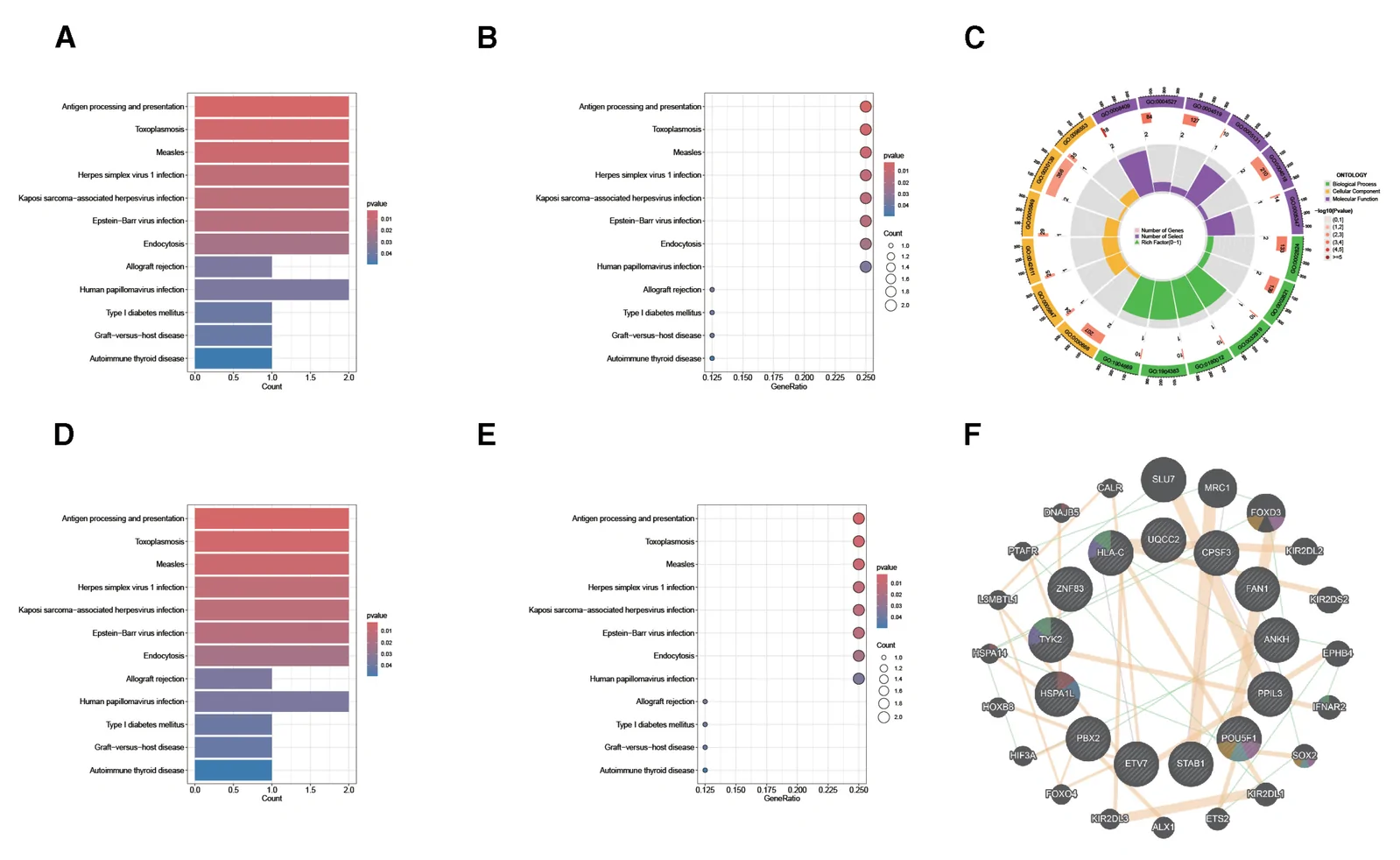
GO analysis, KEGG analysis, and protein-protein interaction network bar chart of GO enrichment analysis for intersecting genes: this bar chart displays the enrichment of intersecting genes in different GO categories (biological processes, cellular components, and molecular functions) through GO enrichment analysis. The height of each bar represents the number of genes enriched in each GO term, and the color indicates the level of significance, with darker bars representing higher enrichment and smaller *P*-values. The chart shows significant enrichment of genes related to rheumatoid arthritis (RA) and acute sinusitis (AS) in several biological processes such as immune response and extracellular matrix regulation (A). Bubble Chart of GO enrichment for intersecting genes: this bubble chart shows the enrichment of intersecting genes across different GO terms. The size of each bubble represents the number of genes enriched in each GO term, and the color intensity reflects the significance of the GO term (*P*-value). This chart helps us intuitively understand the relative distribution and significance of intersecting genes in the categories of biological processes, cellular components, and molecular functions (B). GO circle plot for intersecting genes: the GO circle plot presents a more intuitive representation of the enrichment of intersecting genes across different GO categories. Each ring represents a GO category, with the size and color of the ring indicating the proportion and number of enriched genes, respectively. The color intensity reflects the *P*-value. This plot further reveals the distribution of genes associated with RA and AS in biological processes, molecular functions, and other domains (C). Bar chart of KEGG enrichment analysis for intersecting genes: this bar chart displays the enrichment of intersecting genes in KEGG pathways. Each bar represents a significantly enriched KEGG pathway, with the height of the bar representing the number of genes enriched in that pathway. The color of the bar reflects the significance of the *P*-value, with darker bars indicating higher enrichment and smaller *P*-values. The chart reveals significant enrichment of genes related to RA and AS in several important biological pathways such as immune response, antigen processing and presentation, and viral infection (D). Bubble chart of KEGG enrichment for intersecting genes: this bubble chart displays the relationship between the gene ratio (GeneRatio) and the *P*-value for each KEGG pathway. The size of each bubble indicates the number of genes enriched in each pathway, and the color intensity reflects the significance of the enrichment, with darker bubbles representing smaller *P*-values. This chart allows us to visually observe which KEGG pathways are significantly enriched in RA and AS, providing important clues for further research (E). Protein-protein interaction network based on intersecting genes: this figure shows the protein-protein interaction (PPI) network based on 18 intersecting genes (ETV7, STAB1, TYK2, CPSF3, UQCC2, ANKH, etc.). Each circle represents a gene or protein, with the size of the circle related to its connectivity in the network. Larger circles indicate greater centrality in the network. The lines between circles represent interactions between genes, with the thickness of the lines reflecting the strength of these interactions, where thick lines represent known direct interactions and thin lines represent predicted potential interactions. Different colors of the circles represent different gene functional types; for example, green circles represent immune response-related genes, while yellow circles represent genes associated with cell proliferation and differentiation. This network was generated based on the GeneMANIA database (GeneMANIA, 2021 version) and incorporates results from gene co-expression, functional predictions, and genetic variation analysis, revealing the potential roles of these genes in biological processes such as immune response and cell regulation (F).

#### 3.5.2. GO bubble plot of intersection genes

The bubble plot further reveals the distribution of genes related to RA and AS in biological functions by showing the gene ratio (GeneRatio) and *P*-value for each GO term. The size of the bubble corresponds to the number of genes, and the color intensity indicates the significance of enrichment, allowing for a visual representation of the relative importance and degree of enrichment of each GO term (Fig. 5B).

#### 3.5.3. GO circle plot of intersection genes

The GO circle plot provides a detailed visual presentation for each GO term, combining the gene ratio, gene number, and *P*-value. This plot allows us to better understand the distribution of intersection genes in different GO categories (such as biological processes, cellular components, and molecular functions), highlighting several important biological processes, such as immune response, cell proliferation, and antigen processing (Fig. 5C).

#### 3.5.4. *Kyoto encyclopedia of genes and genomes (KEGG Pathway Enrichment Bar Plot of Intersection Genes*)

This bar plot of intersection genes Each bar represents a significantly enriched KEGG pathway, with the height of the bar indicating the number of genes enriched in that pathway and the color representing the *P*-value significance. The plot helps to identify the key biological processes and diseases involved in RA and AS, such as antigen processing and presentation, toxic degradation, and HIV infection (Fig. 5D).

#### 3.5.5. KEGG pathway bubble plot of intersection genes

The bubble plot further displays the relationship between the gene ratio (GeneRatio) and *P*-value for each KEGG pathway. The size of the bubbles corresponds to the number of enriched genes, and the color intensity indicates the significance of enrichment, with darker bubbles representing smaller *P*-values and more significant enrichment. This chart allowed us to visually understand which KEGG pathways play important roles in both diseases and how genes are distributed within these pathways (Fig. 5E).

#### 3.5.6. Protein-protein interaction network of intersection genes

Based on the GeneMANIA tool was created for 18 intersection genes, including *ETV7*, *STAB1*, *TYK2*, *CPSF3*, *UQCC2*, and *ANKH*. Network analysis revealed multiple direct and predicted interactions among these genes. Genes such as *ETV7*, *STAB1*, *TYK2*, and *HLA-C* were identified as central nodes, indicating their crucial roles in the immune response and cell regulation. Interactions in the network were based on co-expression analyses, functional predictions, and genetic variation data. Thin lines represent predicted interactions, while thick lines represent known direct protein-protein interactions. Functional annotation analysis showed that these genes are associated with biological processes such as “de novo protein folding,” “stem cell population maintenance,” and “immune response.” immune response. immune response. Additionally, top-ranking genes such as *SLU7*, *MRC1*, and *FOXD3* play significant roles in immune modulation and cell proliferation. The data analysis indicated that the synergistic action of these genes provides new insights into the study of immune diseases such as RA (Fig. 5F).

## 4. Discussion

This study used TSMR analysis to explore the genetic relationship between RA and AS, with a particular focus on investigating whether RA is a causal factor for AS. By analyzing publicly available GWAS datasets, we identified several SNPs associated with both RA and AS, providing important insights into the genetic overlap between the 2 diseases.

Our results suggest that RA may be an independent risk factor for AS, with IVW analysis supporting this causal relationship. Specifically, the IVW analysis showed an OR of 1.275 (95% CI: 1.074–1.512, *P* = .005), indicating that the occurrence of RA increases the risk of developing AS. This relationship was further supported by other MR methods such as MR-Egger and weighted median, although the *P*-values did not reach statistical significance in these cases. These findings align with previous epidemiological studies suggesting that immune dysregulation in RA may make individuals more susceptible to infections of the upper respiratory tract, such as sinusitis.^[14]^

In contrast, reverse MR analysis (exploring whether AS leads to the development of RA) did not show significant results. IVW analysis revealed an OR of 0.991 (95% CI: 0.955–1.029, *P* = .642), suggesting that AS does not have a significant causal effect on the development of RA. These findings are consistent with our hypothesis that RA may be a causal factor of AS.

GO and KEGG pathway analyses further confirmed the importance of the intersecting genes in immune response and cell proliferation pathways. Genes such as *ETV7*, *TYK2*, and *ANKH* play crucial roles in immune regulation and inflammation and may be involved in the pathogenic mechanisms of both RA and AS through the immune system.^[11]^

Although some of our findings align with those in the literature, there are discrepancies with some previous studies. First, the effect sizes of certain genes in RA and AS differed, particularly for some low-risk genes (e.g., *TYK2*, *CPSF3*, and *UQCC2*), whose effect sizes were inconsistent across different MR methods. For example, the low-risk effect of *TYK2* on RA was not clearly confirmed in relation to AS in the present study. Previous studies suggest that *TYK2* may play a significant role in other immune system diseases; however, in this study, these genes had minimal effects on AS. This discrepancy could be attributed to differences in the population, disease spectrum, and environmental factors.^[10]^

Additionally, certain genes, such as *HLA-C* and *POU5F1*, showed positive values for their low-risk effects in AS, which differs from the established understanding of their roles in immune regulation. Some studies suggest that *HLA-C* is closely related to immune response and antigen presentation.^[15]^ We speculate that this difference could be related to population genetic differences, sample selection, and the immune mechanisms of AS, as these genes may have varying effects across different populations.

The reasons for these inconsistencies may be due to several factors. First, differences in the study samples might be a major factor influencing inconsistent effect estimates. The GWAS data used in this study came primarily from European populations, whereas some existing studies came from different populations or regions. Genetic diversity between populations can lead to different expression levels of the same gene in different groups.^[11]^ Therefore, future studies should expand the sample size and include data from multiple populations to validate the role of these genes across different groups.

Second, although we excluded genes with significant pleiotropy in the analysis, the complexity of the immune system may have led to certain genes regulating multiple immune pathways simultaneously, which could influence their effects on different diseases. The pleiotropic nature of the immune system is an area worthy of further investigation, and future research could delve deeper into the multiple roles of these genes in both RA and AS and their shared effects on disease.

Epidemiological data on RA show that RA is a common autoimmune disease, primarily affecting individuals aged 30 to 50, with a higher prevalence in women.^[15]^ Its pathogenesis involves genetic susceptibility, abnormal activation of the immune system, and environmental factors. In our study, the importance of immune-related genes in the genetic overlap between RA and AS further validated the crucial role of immune response in the pathogenesis of both diseases. AS is a common infectious disease of the upper respiratory tract, often caused by viral or bacterial infections^[16]^ (AS in daily clinical practice). However, owing to immune system dysregulation, patients with RA may be more susceptible to pathogens that cause AS.^[17]^ Epigenetic modifications and genetic factors have been shown to jointly influence autoimmune disease development, particularly in RA.^[18]^Our study further highlights the interaction of immune genes between RA and AS, and provides new insights into the immunological mechanisms of these diseases.

## 5. Conclusion

This study used MR analysis to investigate the causal relationship between RA and AS and combined Venn diagram analysis, GO analysis, and KEGG pathway analysis to reveal the genetic overlap of multiple immune-related genes between the 2 diseases. Our findings suggest that RA may be an independent risk factor for AS, with immune system genes playing key roles in the pathogenesis of both the diseases.

Through IVW and MR-Egger analyses, we confirmed the joint role of genes such as *ETV7*, *STAB1*, and *ANKH* between RA and AS, which are involved in the immune response and cell cycle processes. GO and KEGG analyses further validated the importance of these genes in immune system regulation and antigen presentation, thereby supporting a potential genetic overlap between RA and AS.

### 5.1. Clinical implications

Given the observed causal effect of RA on AS (OR 1.28), clinicians should consider proactive screening for sinonasal symptoms in RA patients with RA, especially those with active or poorly controlled disease. Targeting shared IL-17–mediated pathways may offer dual benefits for joint and upper airway inflammation. Future prospective cohorts are needed to validate these findings in diverse ancestries.

Although this study is consistent with the existing literature in many aspects, we also observed some discrepancies with previous studies, which could be attributed to sample population differences, immune mechanism variations, and the pleiotropic effects of other genetic factors. Therefore, future research should be conducted with larger sample sizes, across different populations, and considering gene-environment interactions to verify these findings.

### 5.2. Limitations and future directions

This study had several limitations. First, the GWAS datasets used were primarily from European populations, which may have introduced population differences that led to varying genetic effects. Future research should validate these findings across multiple populations to ensure broad applicability of the results. Second, although we excluded the interference of environmental factors through MR, other genetic factors and gene-environment interactions may still affect the accuracy of the results. Future studies should explore gene-environment interactions, especially the impact of infection history and lifestyle factors on the immune system.

## Author contributions

**Methodology:** Rui Hu.

**Data curation:** Yunwang Zhang.

**Resources:** Yunwang Zhang.

**Software:** Yunwang Zhang.

**Visualization:** Yunwang Zhang.

**Investigation:** Yi Zeng.

**Supervision:** Keqiu Dong.

**Validation:** Zhongyun Li.

**Formal analysis:** Jianping Tao.




