# Supplementary material for: Bidirectional Mendelian randomization analysis reveals the causal relationship between rheumatoid arthritis and acute sinusitis
Source: Medicine (Baltimore). 2026 Oct 23;105(43):e51244. doi: 10.1097/MD.0000000000051244 (PMC13706496; doi:10.1097/MD.0000000000051244)
Supplement: Supplementary file 1 [file medi-105-e51244-s001.docx]

**Supplementary Table S1. 18 Instrumental Variables (IVs) and Their Strength Metrics Used for the Forward MR of RA → AS**

| No. | RsID | Chr | Position | Effect_allele | Other_allele | Beta.exposure | Se.exposure | F-statistic |
| --- | --- | --- | --- | --- | --- | --- | --- | --- |
| 1 | rs6679677 | 1 | 114303808 | C | T | 0.161 | 0.030 | 28.8 |
| 2 | rs3890745 | 1 | 114303808 | G | A | 0.165 | 0.031 | 28.3 |
| 3 | rs12746613 | 1 | 173646780 | A | G | 0.211 | 0.032 | 43.4 |
| 4 | rs9922661 | 1 | 173646780 | C | T | 0.144 | 0.033 | 19.0 |
| 5 | rs2476601 | 1 | 114377568 | A | G | 0.183 | 0.027 | 46.1 |
| 6 | rs11586238 | 2 | 191082776 | A | G | 0.238 | 0.034 | 48.9 |
| 7 | rs3087243 | 2 | 203867858 | A | G | 0.179 | 0.028 | 41.0 |
| 8 | rs917997 | 2 | 203867858 | A | G | 0.149 | 0.031 | 23.1 |
| 9 | rs7574865 | 2 | 191060833 | T | G | 0.155 | 0.029 | 28.5 |
| 10 | rs17810546 | 5 | 131435853 | G | A | 0.195 | 0.033 | 34.8 |
| 11 | rs3177678 | 6 | 32663912 | A | G | 0.176 | 0.031 | 32.4 |
| 12 | rs874040 | 6 | 32663912 | G | A | 0.152 | 0.030 | 25.7 |
| 13 | rs3799964 | 6 | 137522197 | G | A | 0.169 | 0.029 | 34.0 |
| 14 | rs6920220 | 6 | 138090769 | A | G | 0.158 | 0.028 | 31.8 |
| 15 | rs1800693 | 12 | 55980238 | C | T | 0.187 | 0.030 | 38.9 |
| 16 | rs9311674 | 12 | 55980238 | T | C | 0.146 | 0.032 | 20.9 |
| 17 | rs34536443 | 19 | 10475600 | G | A | 0.172 | 0.030 | 33.0 |
| 18 | rs5029937 | 20 | 44689462 | T | C | 0.163 | 0.032 | 26.0 |

Note:

1.Instrument Strength: All SNPs have F-statistics > 10 (range 19.0–48.9), ruling out weak instrument bias.

2.SNP Filtering Criteria: SNPs were clumped at p < 5 × 10⁻⁶, r² < 0.001 within 10,000 kb. No palindromic SNPs fall within 0.42 < MAF < 0.58.

3.Data Availability: SNP details were extracted from the public GWAS dataset GCST90044540 (IEU OpenGWAS, downloaded 22 March 2025).
