# Supplementary material for: Bidirectional Mendelian randomization analysis reveals the causal relationship between rheumatoid arthritis and acute sinusitis
Source: Medicine (Baltimore). 2026 Oct 23;105(43):e51244. doi: 10.1097/MD.0000000000051244 (PMC13706496; doi:10.1097/MD.0000000000051244)
Supplement: Supplementary file 2 [file medi-105-e51244-s002.docx]

**Supplementary Table S2. MR-PRESSO Validation Results for RA → AS (18 IVs)**

| Test type | Global test | Outlier test | MR-PRESSO-corrected OR |
| --- | --- | --- | --- |
| Statistic | 1.92 | — | 1.28 |
| P-value | 0.14 | >0.05 | — |
| Interpretation | No overall pleiotropic bias | No significant outlier SNP | Consistent with IVW estimate |
